# Percutaneous endomyocardial ablation as a septal reduction strategy in symptomatic obstructive hypertrophic cardiomyopathy: A 20-year single-center observational experience

**DOI:** 10.1016/j.hrcr.2026.07.028

**Published:** 2026-08-05

**Authors:** Aoife Casey, John McCormick, David Keane

**Affiliations:** Department of Cardiac Arrhythmia, St. Vincent’s University Hospital, Dublin, Ireland

**Keywords:** Hypertrophic, Obstructive, Cardiomyopathy, Septal, Reduction, Ablation, Cryoablation, Endomyocardial, Radiofrequency

**Key Teaching Points**

• Long-term outcome data for endomyocardial ablation in hypertrophic obstructive cardiomyopathy are limited.
• This 20-year single-center experience suggests that endomyocardial ablation can provide sustained reduction in left ventricular outflow tract gradients and symptomatic improvement in selected patients.
• Conduction disturbances may occur early in the learning curve, but procedural evolution and improved targeting strategies may reduce these complications.

## Introduction

Hypertrophic cardiomyopathy (HCM) is a genetic myocardial disorder characterized by unexplained left ventricular hypertrophy and a prevalence of approximately 1:500 in the general population.^1^^,^^2^ Dynamic left ventricular outflow tract (LVOT) obstruction occurs in up to 70% of patients under resting or provoked conditions.^3^ Obstruction is multifactorial and results from septal hypertrophy, altered mitral valve geometry, and systolic anterior motion of the mitral valve.^3^^,^^4^ Although systolic anterior motion contributes to obstruction, it is not invariably the sole mechanism.

Initial management consists of nonvasodilating β-blockers, non-dihydropyridine calcium channel blockers, disopyramide, and, more recently, cardiac myosin inhibitors.^4^^,^^5^ In patients with persistent New York Heart Association (NYHA) functional class III–IV heart failure symptoms and LVOT gradient ≥50 mm Hg despite optimal medical therapy, septal reduction therapy (SRT) is recommended.^5^^,^^6^

Surgical myectomy remains the gold standard in experienced centers.^6^ Transcoronary ablation of septal hypertrophy (TASH) is an alternative in selected patients; however, suitability depends on the septal perforator anatomy. Contemporary high-volume centers report that approximately 5% of patients are unsuitable for TASH because of unfavorable coronary anatomy.^7^^,^^8^

Endomyocardial ablation (EMA) has been explored as a catheter-based technique targeting hypertrophied septal myocardium directly and independently of coronary anatomy.^9^, ^10^, ^11^, ^12^ The procedure was first described by Lawrenz and Kuhn^13^ as a novel catheter-based therapeutic approach for patients with severe obstructive hypertrophic cardiomyopathy. Subsequent reports, particularly in pediatric populations, demonstrated encouraging hemodynamic and clinical outcomes, prompting further evaluation of the technique and contributing to the development of the currently available literature on EMA.^14^^,^^15^

However, published data remain limited, heterogeneous, and largely observational. This study reports our 20-year institutional experience with EMA, focusing on long-term outcomes, procedural limitations, and patient selection rather than on feasibility alone.

## Methods

### Study design

This study is a single-center retrospective observational case series conducted between March 2005 and August 2024. The objective was to evaluate the clinical and hemodynamic outcomes of percutaneous transvalvular EMA in patients with symptomatic obstructive HCM. Institutional approval was obtained, and all patients provided written informed consent for the procedure and longitudinal follow-up.

### Patient selection

8 consecutive patients with obstructive HCM were included (see Table 1). Eligibility criteria were as follows:

• NYHA functional class III or IV heart failure symptoms
• LVOT gradient ≥50 mm Hg at rest or with physiological provocation
• Maximal septal wall thickness ≥18 mm
• Persistent symptoms despite maximally tolerated medical therapy, including nonvasodilating β-blockers, non-dihydropyridine calcium channel blockers, and disopyramide, where appropriate
• All patients underwent comprehensive transthoracic echocardiography before intervention. Invasive hemodynamic measurements were obtained at the time of the procedure.

**Table 1:** Baseline clinical characteristics and acute hemodynamic outcomes

Patient no.	Age (y)	LVEF (%)	Syncope	Medical therapy at the time of procedure	Implantable cardiac defibrillator	LVOT pre procedure (mm Hg)	Setal thickness pre procedure (mm)	LVOT post procedure (mm Hg)	Setal thickness post procedure (mm)
1	60	70	No	Bisoprolol 5 mg	Yes	70	21	15	19
2	68	77	No	Bisoprolol 7.5 mg, verapamil SR 120 mg	Yes	126	23	160	29
3	42	75	No	Bisoprolol 2.5 mg	Yes	100	19	40	19
4	62	45	No	Bisoprolol 1.25 mg, verapamil SR 120 mg	Yes	81	28	21	21
5	79	55	No	Bisoprolol 5 mg	Yes	81	21	30	19
6	59	55	No	Bisoprolol 7.5 mg	Yes	95	22	3	18
7	58	70	No	Bisoprolol 3.75 mg	Yes	180	28	40	28
8	70	60	No	Bisoprolol 10 mg, verapamil SR 120 mg	Yes	190	18	116	16

Preprocedural measurements were measured within 3 mo before the procedure. Postprocedure data were collected immediately after the procedure in the catheterization laboratory. All LVOT values presented are resting gradients.

LVEF = left ventricular ejection fraction; LVOT = left ventricular outflow tract; SR = sustained release.

### Multidisciplinary evaluation and rationale for EMA selection

All cases were reviewed in a multidisciplinary structural heart team meeting involving electrophysiology, cardiology, imaging specialists, and cardiac surgery.

EMA was considered in patients for whom conventional SRT was deemed high risk or less suitable. Factors contributing to the selection of EMA included advanced age, significant comorbidities increasing surgical risk, anatomical complexity, perceived elevated operative risk, or patient preference after detailed counseling regarding available treatment options.

EMA was not offered as first-line SRT in preference to surgical myectomy or TASH, where those therapies were considered appropriate and feasible.

### Procedural technique

3 patients underwent cryothermal EMA in 2005, while 5 patients subsequently underwent radiofrequency (RF) EMA between 2012 and 2024, reflecting the evolution of institutional practice and available technology.

Procedures were performed under conscious sedation in the early cryoablation cases and under general anesthesia in all RF ablation cases. Continuous invasive hemodynamic monitoring was performed throughout each procedure using simultaneous left ventricular and aortic pressure recordings to assess dynamic LVOT obstruction.

Transseptal and/or retrograde transaortic approaches were used depending on anatomical considerations.

Ablation lesions were delivered to the basal interventricular septum under fluoroscopic and electroanatomic mapping guidance in RF ablation cases. In cryoablation cases, lesions were applied using transvalvular catheter positioning guided by fluoroscopy.

Acute procedural success was predefined as a reduction of greater than 50% in the invasive LVOT gradient at the conclusion of the procedure.

### Cryoablation system

As previously reported,^9^ cryoablation was performed in 3 patients (patients 1–3). Patient 1 underwent ablation via a retrograde transaortic approach using a 7-F, 4-mm-tip cryocatheter. Patients 2 and 3 underwent antegrade transseptal procedures using a 9-F, 8-mm-tip quadripolar cryocatheter with 3- and 2-mm interelectrode spacing (Freezor MAX, CryoCath Technologies Inc., Montreal, Quebec, Canada). Under fluoroscopic guidance, transseptal access to the left atrium was obtained using a 10-F transseptal Mullins sheath (Mullins Introducer Sheath/ Brokenbrough needle, Medtronic, Stockholm, Sweden) via the right femoral vein. This antegrade approach facilitated catheter-septal contact by allowing a parallel electrode orientation with the endocardium of the LVOT, while the retrograde transaortic approach resulted in a more perpendicular electrode orientation. Cryolesions were delivered to the basal interventricular septum under fluoroscopic guidance. 20–32 cryothermal applications, each lasting 240 seconds, were applied to the LVOT septum under fluoroscopic guidance. The mean sustained temperature was −88°C.

### RF ablation system

RF catheter ablation was performed in 5 patients (patients 4–8) using the CARTO 3.5 three-dimensional electroanatomic mapping system (Biosense Webster, Johnson & Johnson MedTech, Irvine, CA). Ablation was performed using an 8-F, 3.5-mm irrigated-tip THERMOCOOL SMARTTOUCH SF catheter (Biosense Webster) with 1-6-2 mm interelectrode spacing.

An exclusive antegrade transseptal approach was used in patients 4 and 5. In patients 6–8, both antegrade transseptal and retrograde transaortic approaches were used according to septal anatomy and catheter stability requirements. Electroanatomic mapping was used to identify the basal septal target region and to minimize proximity to the conduction system by mapping fascicular potentials (Figure 1).

**Figure 1:**
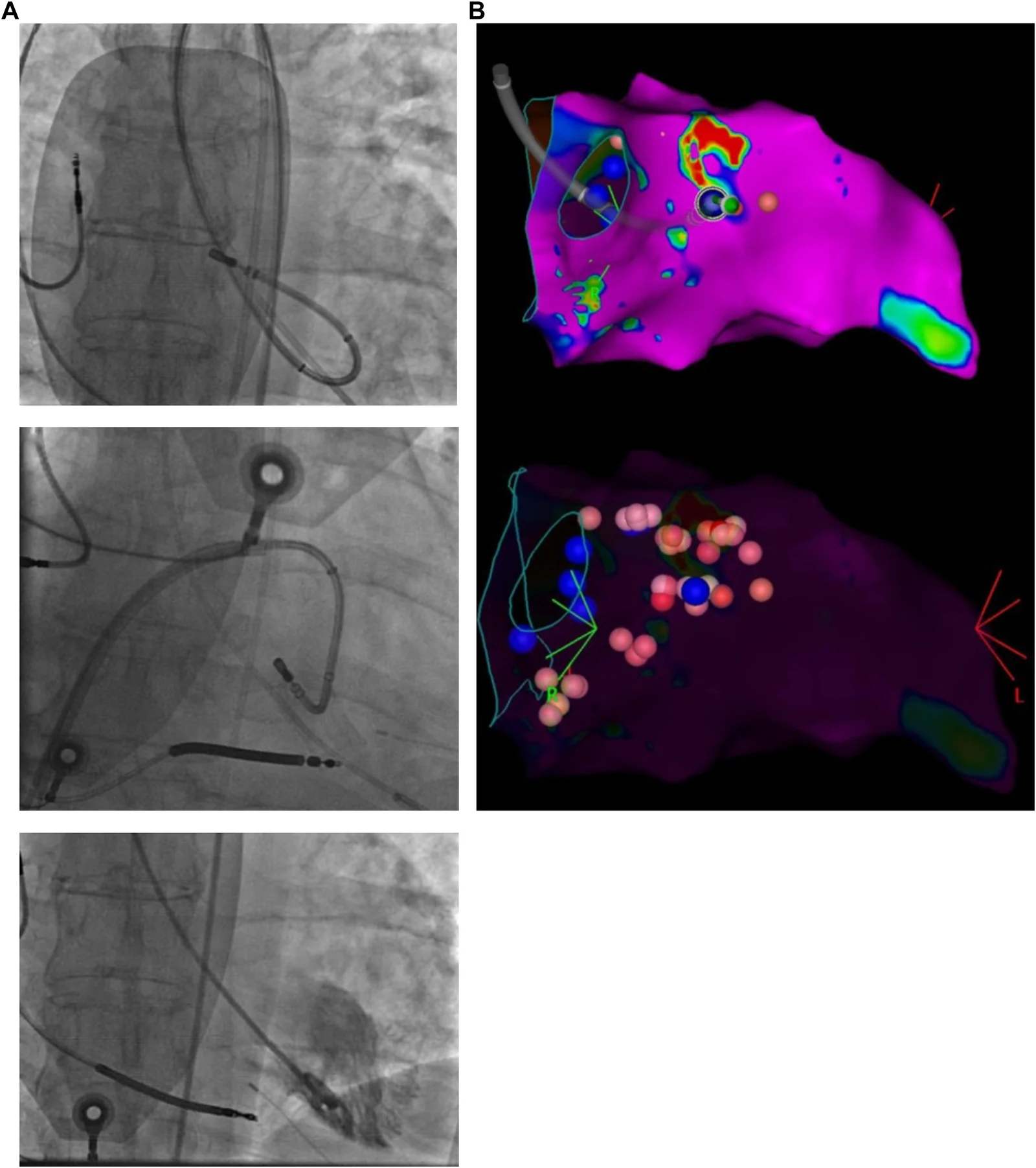
Intraprocedural imaging. **A:** Fluoroscopic images illustrating procedural approaches for endomyocardial ablation. *Top:* Retrograde transaortic approach via the femoral artery with catheter positioning in the left ventricular outflow tract. *Middle:* Antegrade transseptal approach after femoral venous access and interatrial septal puncture. *Bottom:* Left ventriculography demonstrating septal hypertrophy and dynamic subvalvular obstruction. **B:** Electroanatomic voltage mapping and invasive hemodynamic assessment during endomyocardial radiofrequency ablation. 3-dimensional left ventricular endocardial voltage map demonstrating ablation lesion sets (*red tags*) delivered to the basal interventricular septum. *Blue markers* indicate mapped fascicular potentials to guide avoidance of the conduction system.

RF energy was delivered in a point-by-point fashion at a power setting of 40 W for a maximum duration of 120 s/lesion (range 60–120 s/lesion) (Table 2).

**Table 2:** Procedural details

Patient no.	Sedation	Access	Approach	Fluoroscopy time (min)	Ablation applications × duration	Power setting of RFA	Intraprocedural complication	ECG postprocedure
1	Conscious	RFA 10 F	Retrograde transaortic	31.2	32 × 240 s	NA	None	Normal sinus rhythm
2	Conscious	RFV 10 F	Antegrade transseptal	10.7	23 × 240 s	NA	None	Normal sinus rhythm
3	Conscious	RFV 10 F	Antegrade transseptal	13.4	20 × 240 s	NA	None	Normal Sinus Rhythm
4	General	RFV 10 F + RFA 10 F	Antegrade transseptal	14.3	20 × 120 s	40 W	Transient AVB	Ventricular pacing
5	General	RFV 8 F + RFA 8 F	Retrograde + transseptal	NA	15 × 60 s	40 W	Transient AVB	Normal sinus rhythm
6	General	RFA 10 F	Retrograde transaortic	NA	34 × 60 s	40 W	Transient AVB	Normal sinus rhythm
7	General	RFV 8 F + RFA 8 F	Retrograde + transseptal	NA	20 × 120 s	40 W	None	Left bundle branch block
8	General	RFV 8 F + RFA 8 F	Retrograde + transseptal	13.31	20 × 90 s	40 W	None	Left bundle branch block

AVB = atrioventricular block; NA = not available; RFA = right femoral artery; RFV = right femoral vein.

### Follow-up and outcome assessment

Patients were reviewed at 3, 6, and 12 months and annually thereafter. Follow-up assessment included clinical review with NYHA functional class evaluation, transthoracic echocardiography, 12-lead electrocardiography, and implantable cardioverter-defibrillator interrogation, where applicable.

Echocardiographic assessment of LVOT obstruction was performed immediately postprocedure and at each follow-up visit thereafter, using continuous-wave (CW) Doppler interrogation from multiple acoustic windows, including apical 5-chamber, apical long-axis, and right parasternal views, in accordance with contemporary echocardiographic recommendations for HCM. Resting and provoked LVOT gradients were assessed using the modified Bernoulli equation (P = 4 (V2^2^ - V1).

Care was taken to distinguish the LVOT Doppler envelope from mitral regurgitation (MR) signals. LVOT obstruction was identified by its characteristic late-peaking, dagger-shaped CW Doppler profile, whereas MR jets demonstrated earlier onset, higher velocity, and a denser parabolic spectral envelope. Doppler alignment was optimized to avoid contamination of LVOT measurements by MR signals.

Pulsed-wave Doppler was used only for the localization of flow acceleration within the LVOT and not for gradient quantification, as pulsed-wave Doppler may underestimate high-velocity dynamic obstruction. All reported LVOT gradients were derived from CW Doppler (Figure 2).

**Figure 2:**
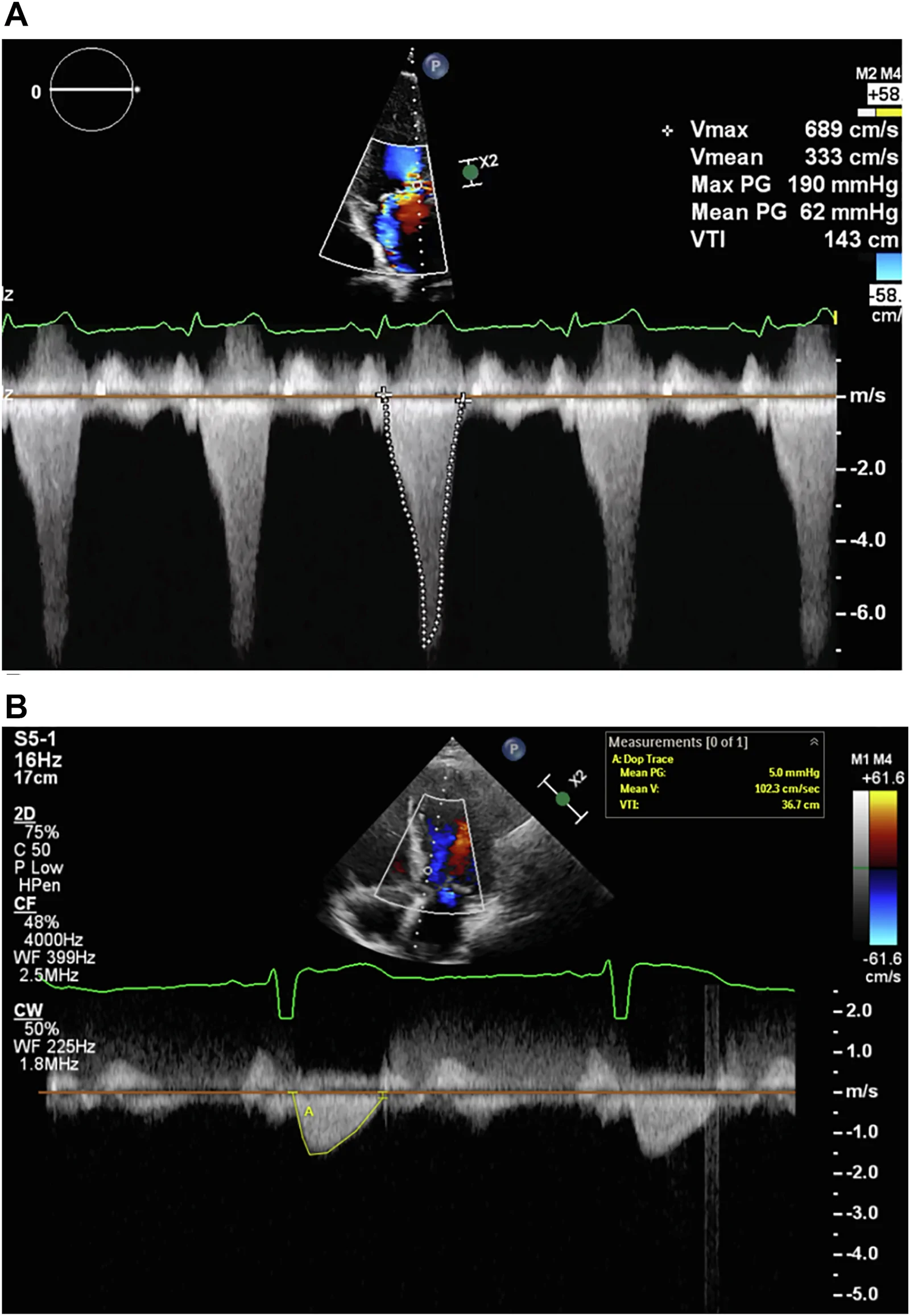
Transthoracic echocardiographic continuous-wave Doppler images from patient 8. **A:** Preprocedural study, demonstrating severe left ventricular outflow tract (LVOT) obstruction with a peak instantaneous gradient of 190 mm Hg. **B:** Transthoracic echocardiography performed 3 months postprocedure showing a marked reduction in LVOT obstruction, with only a trivial residual resting gradient and no hemodynamically significant obstruction. The patient reported complete resolution of exertional symptoms (New York Heart Association functional class I).

The primary hemodynamic end point was the change in LVOT gradient from baseline to immediately postprocedure and during long-term follow-up. Secondary end points included change in NYHA functional class and procedural safety outcomes.

The mean follow-up duration was 12.8 years (range 9 months to 19.2 years).

### Statistical analysis

Given the small sample size and retrospective case-series design, statistical analysis was primarily descriptive.

Continuous variables are presented as means ± standard deviations, where appropriate. Given the limited sample size, assessment of normality was not formally performed. Categorical variables are reported as counts and percentages.

Changes in LVOT gradients are described as absolute and relative percentage differences between baseline, immediately postprocedure, and long-term follow-up measurements.

Exploratory paired analysis using the Wilcoxon signed-rank test demonstrated significant reductions in LVOT gradient both immediately after the procedure (115.4 ± 46.2 mm Hg vs 53.1 ± 55.1 mm Hg; *P* = .016) and at the most recent follow-up (115.4 ± 46.2 mm Hg vs 30.1 ± 26.2 mm Hg; *P* = .0078). Results were consistent in sensitivity analyses using paired *t* tests (Supplemental Table 1).

## Results

### Baseline characteristics

Between March 2005 and August 2024, 8 patients (7 male, 1 female) underwent percutaneous EMA for symptomatic obstructive HCM. The mean age at intervention was 62 years (range 42–79 years). All patients had NYHA functional class III a heart failure symptoms baseline despite maximally tolerated medical therapy.

The mean preprocedural invasive LVOT gradient was 115.6 ± 43.2 mm Hg.

### Acute hemodynamic outcomes

#### Cryoablation cohort (patients 1–3)

Baseline invasive LVOT gradients in the cryoablation cohort were 70, 126, and 100 mm Hg, respectively.

2 patients demonstrated immediate reduction in LVOT gradient after cryoablation. In contrast, patient 2 demonstrated an immediate postablation gradient of 160 mm Hg (an increase from the baseline value of 126 mm Hg), likely reflecting transient edema and incomplete lesion formation rather than the absence of obstruction. This case was therefore classified as procedural failure in terms of acute hemodynamic success. No procedural complications occurred.

#### RF ablation cohort (patients 4–8)

Baseline invasive LVOT gradients in the RF cohort were 81, 81, 95, 180, and 190 mm Hg, respectively.

Immediate postprocedural gradients were 21, 30, 3, 40, and 116 mm Hg, respectively. An acute gradient reduction of greater than 50% was achieved in 6 of 8 patients overall.

2 patients (patients 2 and 8) demonstrated a limited acute hemodynamic response.

Across the entire cohort, the mean immediate postprocedural LVOT gradient was 53.1 ± 55.1 mm Hg (Table 1).

### Long-term hemodynamic outcomes

At a mean follow-up of 12.8 years (range 9 months to 19.2 years), the mean LVOT gradient across the cohort was 21.5 ± 28.0 mm Hg at the most recent follow-up, representing an average relative reduction of 62% compared with baseline values.

In the cryoablation cohort, long-term gradients were 58 mm Hg (patient 1), 44 mm Hg (patient 2), and 50 mm Hg (patient 3) at the most recent follow-up. No patient demonstrated complete abolition of the LVOT gradient. Gradients were reduced to low or clinically nonsignificant dynamic levels in most cases.

In the RF cohort, sustained gradient reduction was observed in the majority of patients, although variability between individuals remained (Table 3 and Figure 3).

**Table 3:** Long-term follow-up of LVOT gradients

Patient no.	LVOT pre (mm Hg)	LVOT post (mm Hg)	LVOT at the most recent follow-up (mm Hg)
1	70	15	58
2	126	160	44
3	100	40	50
4	81	21	6
5	81	30	5
6	95	3	4
7	180	40	64
8	190	116	10

The mean follow-up duration was 12.8 y (range 9 mo to 19.2 y).

LVOT = left ventricular outflow tract.

**Figure 3:**
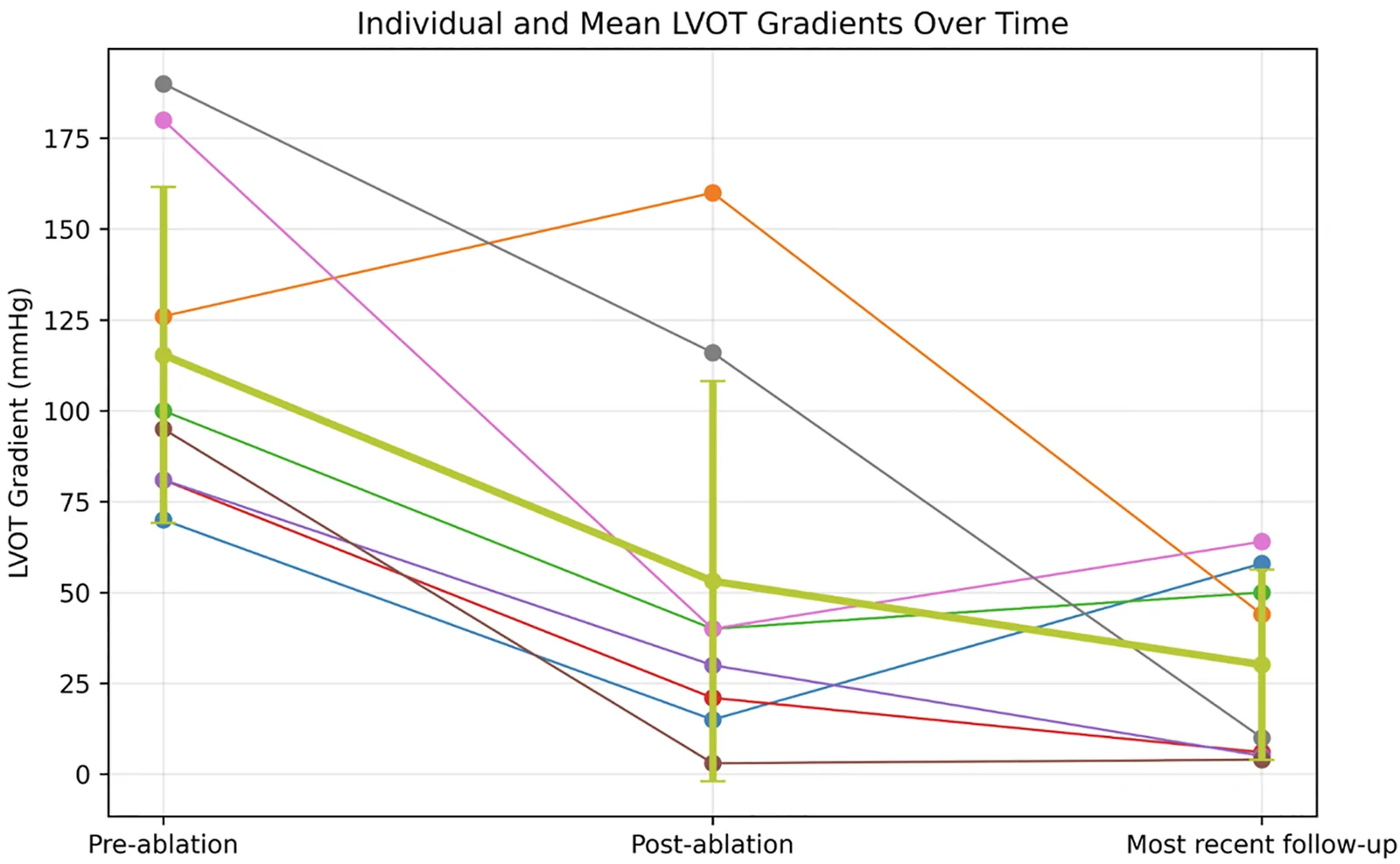
Graph showing the long-term follow-up of left ventricular outflow tract (LVOT) gradients.

All patients in the cohort improved to NYHA functional class I or II at the most recent follow-up. No patient underwent subsequent surgical myectomy.

### Safety outcomes

There were no major adverse cardiovascular or cerebrovascular events during the procedural or follow-up period. Specifically.

• No sudden cardiac deaths
• No appropriate implantable cardioverter-defibrillator therapies
• No strokes

3 patients in the RF cohort developed transient atrioventricular conduction block during the procedure, all of which resolved by completion of the procedure. 2 patients developed left bundle branch block, which resolved at follow-up.

## Discussion

This single-center observational series describes the long-term hemodynamic and clinical outcomes of percutaneous EMA in 8 highly selected patients with symptomatic obstructive HCM. Over a mean follow-up duration of 12.8 years, EMA was associated with sustained reduction in LVOT gradients and improvement in functional status in our cohort, without major adverse cardiovascular events. However, these findings must be interpreted cautiously given the very small cohort, procedural heterogeneity, and limited institutional volume.

EMA achieved a mean relative gradient reduction of 62% at follow-up. Importantly, the therapeutic goal in obstructive HCM is reduction to a clinically nonsignificant dynamic gradient rather than complete abolition of the LVOT pressure difference, which would be physiologically implausible. In this series, gradients were reduced but not eliminated.

3 patients demonstrated limited acute hemodynamic response. This likely reflects the complex and multifactorial nature of LVOT obstruction, including deep septal hypertrophy beyond endocardial lesion depth, incomplete lesion continuity, mitral apparatus contribution, and transient tissue edema. These observations highlight the technical limitations of superficial endocardial energy delivery in a disease characterized by heterogeneous septal morphology. Previous reports have described transient early increases in gradient after RF ablation, likely because of myocardial edema.^11^, ^12^ This phenomenon may explain early hemodynamic variability in our series. Notably, in the patient who demonstrated a postprocedure increase in gradient, long-term follow-up shows a significant reduction over time, supporting the hypothesis of early myocardial edema contributing to a transient increase in gradient.

EMA offers theoretical advantages, including independence from septal coronary anatomy and a percutaneous approach. However, lesion depth is constrained, conduction system proximity remains a concern, and comparative data against established septal reduction therapies are lacking. Surgical myectomy and transcoronary alcohol septal ablation remain the reference standards in appropriately selected patients.

The therapeutic landscape has also evolved with the introduction of cardiac myosin inhibitors, which may reduce the need for invasive septal reduction in some patients. Consequently, the potential role of EMA is likely to remain highly selective and investigational.

### Limitations

This study is limited by its retrospective design, extremely small sample size, 2 different ablation modalities, absence of a control group, and low procedural volume over 2 decades. These factors substantially limit generalizability and preclude conclusions regarding comparative efficacy or safety. Importantly, this series does not establish equivalence in safety or efficacy compared with established septal reduction therapies such as surgical myectomy or TASH, which remain the standard of care in appropriately selected patients.

Intraprocedural imaging with intracardiac echocardiography was not routinely used, and access to cardiac magnetic resonance imaging data for lesion characterization, assessment of myocardial scar burden, and evaluation of septal remodeling or retraction after ablation was limited. As a result, the mechanistic effects of EMA on myocardial substrate and septal morphology could not be systematically assessed.

Interpretation of longitudinal LVOT gradient outcomes is limited by inconsistency in provocation methods across historical echocardiographic studies. Because of the retrospective design and lack of standardized provocation protocols over the study period, comparison of provocable gradients between time points was considered unreliable. Therefore, follow-up analyses were restricted to resting LVOT gradients, which provided a more consistent measure of treatment effect.

We acknowledge that a proportion of the acute hemodynamic improvement observed immediately after ablation may have been attributable to procedure-related septal conduction disturbances, including new left bundle branch block or transient atrioventricular block requiring temporary pacing. In such cases, ventricular dyssynchrony may contribute to a reduction in LVOT obstruction independent of the direct effects of septal ablation. However, the persistence of gradient reduction during long-term follow-up after resolution of transient conduction abnormalities and in the absence of permanent pacing suggests that the observed hemodynamic benefits were not solely attributable to conduction delay and likely reflect a sustained effect of septal substrate modification.

Despite these limitations, this report provides rare long-term follow-up data on EMA in patients with obstructive HCM.

## Conclusion

In this small, highly selected cohort, EMA was associated with sustained LVOT gradient reduction and symptomatic improvement over long-term follow-up. However, given the methodological limitations and availability of established therapies, EMA should be considered investigational pending validation in larger prospective studies.

## Disclosures

The authors have no conflicts of interest to disclose.
