# Supplementary material for: Percutaneous endomyocardial ablation as a septal reduction strategy in symptomatic obstructive hypertrophic cardiomyopathy: A 20-year single-center observational experience
Source: HeartRhythm Case Rep. 2026 Aug 5;12(10):1228–35. doi: 10.1016/j.hrcr.2026.07.028 (PMC13687970; doi:10.1016/j.hrcr.2026.07.028)
Supplement: Supplementary file 1 [file mmc1.docx]

**Supplementary Methods**

**Statistical Analysis**

Given the small sample size and non-normal distribution of LVOT gradient measurements, paired comparisons between baseline and follow-up values were performed using the Wilcoxon signed-rank test. Paired t-tests were additionally conducted as sensitivity analyses. All tests were two-sided, with statistical significance defined as p < 0.05.

**Supplementary Results**

**Supplementary Table S1. Paired Analysis of LVOT Gradient Reduction Following Septal Ablation**

| **Comparison** | **n** | **Baseline LVOT Gradient (mmHg), Mean ± SD** | **Follow-up LVOT Gradient (mmHg), Mean ± SD** | **Paired t-test** | **Wilcoxon signed-rank** |
| --- | --- | --- | --- | --- | --- |
| Pre-procedure vs Immediate Post-procedure | 8 | 115.4 ± 46.2 | 53.1 ± 55.1 | t = 3.62, p = 0.008 | p = 0.016 |
| Pre-procedure vs Most Recent Follow-up | 8 | 115.4 ± 46.2 | 30.1 ± 26.2 | t = 4.93, p = 0.0017 | p = 0.0078 |

**Supplementary Text**

Paired analysis demonstrated a significant reduction in LVOT gradients immediately following septal ablation and at long-term follow-up. For the comparison between baseline and immediate post-procedural LVOT gradients (n = 8), the mean gradient decreased from 115.4 ± 46.2 mmHg to 53.1 ± 55.1 mmHg. This reduction was significant by paired t-test (t = 3.62, p = 0.008) and Wilcoxon signed-rank test (p = 0.016).

At most recent follow-up (n = 8), the mean LVOT gradient decreased further to 30.1 ± 26.2 mmHg. This reduction remained significant by paired t-test (t = 4.93, p = 0.0017) and Wilcoxon signed-rank test (p = 0.0078).
